# Reviewer acknowledgement 2013

**DOI:** 10.1186/1756-3305-7-49

**Published:** 2014-01-29

**Authors:** Chris Arme

**Affiliations:** 1 Parasites & Vectors, 236 Grays Inn Road, London WC1X 8HB, UK

## Abstract

**Contributing reviewers:**

*Parasites & Vectors* would like to thank all reviewers who have contributed to the journal in 2013.

## 

Mohammad Yazid Abdad

Papua New Guinea

Nicole Achee

United States

Alvaro Acosta-Serrano

United Kingdom

Ishag Adam

Sudan

Yaw Afrane

Kenya

Nazaire Aizoun

Benin

Daniel Ajzenberg

France

Martin Akogbeto

France

Mohammad Shafiul Alam

Bangladesh

Gema Alama Bermejo

United States

Samer Alasaad

Spain

Marco Albonico

Italy

Alexander Mathis

Switzerland

Silvia Alfieri

Brazil

Arshad Ali

United States

Ibne Ali

United States

David Allred

United States

Igor Almeida

United States

Hesham Al-Mekhlafi

Malaysia

Cristina Alonso-Vega

Bolivia

Bulent Alten

Turkey

Cosme Alvarado-Esquivel

Mexico

Gema Alvarez Garcia

Spain

Stephen Ambu

Malaysia

John Anderson

United States

Andrey Andrade

Brazil

Renato Andreotti

Brazil

Anges Yadouleton

Benin

Daniela Antolova

Slovakia

Christophe Antonio-Nkondjio

Cameroon

Peter Armbruster

United States

Marina Ascunce

United States

Alex Asidi

United Kingdom

Hasan Tarik Atmaca

Turkey

Stephen Attwood

China

Herbert Auer

Austria

Stuart Auld

United Kingdom

Tatjana Avsic-Zupanc

Slovenia

Diego Ayala

France

Roseric Azondekon

Benin

Leïla Bagny Beilhe

Cameroon

Venugopal Balakrishnan

Malaysia

Carsten Balczun

Germany

Gad Baneth

Israel

Fu Baoquan

China

Imrich Barak

Slovakia

Iain Barber

United Kingdom

Maria Dolores Bargues

Spain

Maria-Gloria Basanez

United Kingdom

Paul Bates

United Kingdom

Relja Beck

Croatia

Jerzy Behnke

United Kingdom

John Beier

United States

Staffan Bensch

Sweden

Robert Bergquist

Sweden

Eliana Bergter

Brazil

Rasa Bernotiene

Lithuania

Federica Berrilli

Italy

Paul Bessell

United Kingdom

Bernard Bett

Kenya

Peng Bi

Australia

Peter Billingsley

United States

Samuel Black

United States

Damer Blake

United Kingdom

Isabel Blasco-Costa

Spain

Brad Blitvich

United States

Moses Bockarie

United Kingdom

Pascal Boireau

France

Ken Boorom

United States

Ali Bouattour

Tunisia

Michel Boussinesq

France

Jérémy Bouyer

Senegal

Dwight Bowman

United States

Janette Bradley

United Kingdom

Marieta Braks

Netherlands

Alejandra Bravo

Mexico

Daniel Bray

United Kingdom

Reginaldo Brazil

Brazil

Klaus Brehm

Germany

Edward Breitschwerdt

United States

Emanuele Brianti

Italy

Constança Britto

Brazil

William Brogdon

United States

Basil Brooke

South Africa

Fabrizio Bruschi

Italy

Stewart Burgess

United Kingdom

Tom Burkot

Australia

David Buttle

United Kingdom

Jo Cable

United Kingdom

Cinzia Cantacessi

Australia

Gioia Capelli

Italy

Helene Carabin

United States

Luis Cardoso

Portugal

Pilar Carranza Rosales

Mexico

Mark Carrington

United Kingdom

Dave D. Chadee

Trinidad And Tobago

Kwang Poo Chang

United States

Moh-Seng Chang

Vanuatu

Theeraphap Chareonviriyaphap

Thailand

Luis Fernando Chaves

Japan

Qijun Chen

China

Xiaoguang Chen

China

Nakul Chitnis

Switzerland

Bruno Chomel

United States

Wej Choochote

Thailand

Philippe Christe

Switzerland

Donato Cioli

Italy

Edwin Claerebout

Belgium

Jesper Hedegaard Clausen

Denmark

Archie Clements

Australia

Maureen Coetzee

South Africa

Justin Cohen

United States

Anna Cohuet

France

Gerald Coles

United Kingdom

Esther Collantes-Fernandez

Spain

Jan Conn

United States

Bob Cooper

Australia

Vincent Corbel

Benin/ France

Margarita Correa

Colombia

Jean Tenena Coulibaly

Cote D'ivoire

Janet Cox-Singh

United Kingdom

Clemente Capasso

Italy

Giuseppe Cringoli

Italy

Isra Cruz

Spain

Cristina Cutillas

Spain

Cyrille Czeher

Niger

Alexandre da Silva

United States

Ary Gomes da Silva

Brazil

Filipe Dantas-Torres

Brazil

Jonathan Darbro

Australia

Frédéric Darriet

France

Murari Das

Nepal

Siddhartha Das

United States

Tim Day

United States

Arnout De Bruin

Netherlands

Jose Batista De Jesus

Brazil

Claudio De Liberato

Italy

Theo De Waal

Ireland

Eleanor Deardorff

United States

Rinki Michelle Deb

United Kingdom

Stijn Deborggraeve

Belgium

Jerome Depaquit

France

Daniel Depledge

United Kingdom

Marketa Derdakova

Slovakia

Kebede Deribe

Ethiopia

Greg Devine

Australia

Angela Di Cesare

Italy

Ibrahima Dia

Senegal

Diawo Diallo

Senegal

Brent Dixon

Canada

Armel Djenontin

Benin

Michael J. Doenhoff

United Kingdom

Martin Donnelly

United Kingdom

Seydou Doumbia

Mali

Gilles Dreyfuss

France

Aifang Du

China

Ana Maria Duarte

Brazil

Margarida Duarte

Portugal

Luc Duchateau

Belgium

Mike Duffy

Canada

Jean-Pierre Dujardin

France

Thelma Dunnebacke

United States

Pi Durand

South Africa

Ravi Durvasula

United States

Georg Duscher

Austria

Naomi Dyer

United Kingdom

Rebecca Eisen

United States

Kamal El Bissati

United States

Armin Elbers

Netherlands

John Ellis

Australia

Dia-Eldin Elnaiem

United States

Hany Elsheikha

United Kingdom

Aidan Emery

United Kingdom

David Engman

United States

Keeseon Eom

Korea South

Ozge Erisoz Kasap

Turkey

Agustin Estrada-Pena

Spain

Chia-Kwung Fan

Taiwan

Chafika Faraj

Morocco

Leonardo Farias

Brazil

Robert Farkas

Hungary

Asghar Fazaeli

Iran

Yaoyu Feng

China

Andy Fenton

United Kingdom

Heather Ferguson

United Kingdom

Ezio Ferroglio

Italy

Ivan Fiala

Czech Republic

Julio V. Figueroa

Mexico

Robert Finn

United Kingdom

Peter Fischer

United States

Jaroslav Flegr

Czech Republic

Fernando Ivan Flores

Mexico

Monica Florin-Christensen

Argentina

Gabor Foldvari

Hungary

Desmond Foley

United States

Dina Fonseca

United States

Albin Fontaine

France

Didier Fontenille

France

Jeremy Foster

United States

Laura Fraczek

United States

Michel Franc

France

Antonio Frangipane Di Regalbono

Italy

Frits Franssen

Netherlands

Mark Freeman

Malaysia

Simone Freitas

Brazil

Michael French

United Kingdom

Paula Gabriela

Argentina

Surendra Gakhar

India

Eunice Galati

Brazil

Ma. de la Luz Galvan-Ramirez

Mexico

Jean-Charles Gantier

France

Xin Gao

United States

Teresa Gárate

Spain

Joao Luis Garcia

Brazil

Allison Gardner

United States

Giuliano Gasperi

Italy

Robin Gasser

Australia

Michael Gaunt

United Kingdom

Timothy Geary

Canada

Peter Geldhof

Belgium

Claudio Genchi

Italy

David George

United Kingdom

Peter Gething

United Kingdom

Annunziata Giangaspero

Italy

Wendy Gibson

United Kingdom

Lucy Gilbert

United Kingdom

Julia Gillhuber

Germany

John Gimnig

United States

Geoffrey Gimonneau

Burkina Faso

Nicholas Golding

United Kingdom

Yara Miranda Gomes

Brazil

David Gorla

Argentina

Nicodem James Govella

Tanzania

Daniel Grabner

Germany

Luigi Gradoni

Italy

Giulio Grandi

Sweden

Jeremy Gray

Ireland

Mario Grijalva

United States

Felix Grimm

Switzerland

Edmundo Grisard

Brazil

Libor Grubhoffer

Czech Republic

Felix Guerrero

United States

Monika Gulia

United States

David Gurarie

United States

Rodrigo Gurgel-Goncalves

Brazil

Carlos Gutierrez

Spain

Roy Hall

Australia

Katherine Halliday

United Kingdom

Omar Hamarsheh

Palestinian Territory

Sarah Hamer

United States

Ze-Guang Han

China

Penelope Hancock

United Kingdom

Kurt Hanevik

Norway

Immo Hansen

United States

Ralph Harbach

United Kingdom

David Harley

Australia

David Harrington

Denmark

Kathrin Hartelt

Germany

Ashlie Hartigan

Czech Republic

Mo'awia Hassan

Sudan

Frances Hawkes

United Kingdom

Shenyi He

China

Michelle Helinski

Uganda

Helena Helmby

United Kingdom

Andrew Hemphill

Switzerland

Fiona Henriquez

United Kingdom

Tineke Herremans

Netherlands

Leidi Herrera

Venezuela

Geoff Hide

United Kingdom

Nawal Hijjawi

Jordan

Julian Hillyer

United States

Yousif Himeidan

Sudan

Johan Hoglund

Sweden

Celia Holland

Ireland

Sung-Tae Hong

Korea South

David Hoole

United Kingdom

Petter Hopp

Norway

Sandor Hornok

Hungary

Paul Howll

United States

Min Hu

China

Wanqi Hu

United States

Marc Hübner

Germany

Leon Eklund Hugo

Australia

Jerome Hui

Hong Kong

Christopher Hunter

United States

Hilary Hurd

United Kingdom

Adolfo Ibanez-Justicia

Netherlands

Ralf Ignatius

Germany

Jean-Luc Imler

France

Noboru Inoue

Japan

Mariana Ionita

Romania

Seth Irish

United Kingdom

Peter Irwin

Australia

Akira Ito

Japan

Abdul Jabbar

Australia

Raymond Jacobson

Israel

Thomas G.T. Jaenson

Sweden

Nicolas Jaramillo O

Colombia

Musa Jawara

Gambia

Aaron Jex

Australia

Mario Jiz

Philippines

Maria Vang Johansen

Denmark

Christopher Jones

United Kingdom

Malcolm Jones

Australia

Nick Jonsson

United Kingdom

Deirdre Joy

United States

Kerstin Junker

South Africa

Joseph Kamau

Kenya

Laura Kamenetzky

Argentina

Alex Karagiannis

Switzerland

Panagiotis Karanis

Germany

Christian Kaufmann

Switzerland

Shin-Ichiro Kawazu

Japan

Jennifer Keiser

Switzerland

Louise Kelly-Hope

United Kingdom

Volkhard Kempf

Germany

Malcolm Kennedy

United Kingdom

Noteila Khalid

Sudan

Naveed Khan

Pakistan

Solomon Kibret

Australia

Albert Kilian

Spain

Carsten Kirkeby

Denmark

Bart Knols

Netherlands

Stefanie Knopp

Switzerland

Katherine Kocan

United States

Lizette Koekemoer

South Africa

Jacob Koella

United Kingdom

Constantianus J.M. Koenraadt

Netherlands

Aneta Kostadinova

Bulgaria

Andrew Kotze

Australia

Benjamin Koudou

United Kingdom

Laura Kramer

Italy

Michael Kristensen

Denmark

Oliver Krone

Germany

Jürgen Krücken

Germany

Sanjay Kumar

India

Suresh Kumar

Malaysia

Eliningaya Kweka

Tanzania

Oliver Kwok

United States

Giuseppe La Rosa

Italy

Pierrick Labbé

France

Marcelo Labruna

Brazil

Juan Laclette

Mexico

Patrick Lammie

United States

Robert Lane

United States

Lucie Lantova

Czech Republic

Mauricio Lascano

United States

Karin Lebl

Austria

Tovi Lehmann

United States

Francisco Lemos

Brazil

Meryem Lemrani

Morocco

Christian Lengeler

Switzerland

Michael Levin

United States

Edward Levri

United States

Michael Lewis

United Kingdom

Jun Li

United States

Sheng Li

United States

You-Sheng Liang

China

You-Sheng Liang

China

Marshall Lightowlers

Australia

José Lino-Neto

Brazil

Susan Little

United States

Tom Little

United Kingdom

Tim Littlewood

United Kingdom

Guo-Hua Liu

China

Quan Liu

China

Martin Llewellyn

United Kingdom

Matt Longshaw

United Kingdom

Camila Lorenz

Brazil

Bertrand Losson

Belgium

Shirley Luckhart

United States

Hugo Lujan

Argentina

Dickson Lwetoijera

Tanzania

Candice-Lee Lyons

South Africa

Charles Mackenzie

United States

Kenneth Mackenzie

United Kingdom

Henry Madsen

Denmark

Ricardo Maggi

United States

Silas Majambere

Tanzania

Benjamin Makepeace

United Kingdom

Joanna Makol

Poland

Imna Malele

Tanzania

Naiela Malik

United Kingdom

Richard Malik

Australia

Sylvie Manguin

France

Pablo Maravilla

Mexico

Jorge Diego Marco

Argentina

Jerome Marie

French Polynesia

Christine Maritz-Olivier

South Africa

Francisco J. Márquez

Spain

Pioz Maryline

France

Mauro Marzochi

Brazil

Santiago Mas-Coma

Spain

Daniel Masiga

Kenya

Fekadu Massebo

Ethiopia

Toshiyuki Masuzawa

Japan

Alexander Mathis

Switzerland

Nancy Matowo

Tanzania

Simonetta Mattiucci

Italy

Aaron Maule

United Kingdom

Humphrey Mazigo

Tanzania

Leonard Mboera

Tanzania

Charles Mbogo

Kenya

Jere Mcbride

United States

Donald P McManus

Australia

Samantha McNulty

United States

Paul McVeigh

United Kingdom

Jolyon Medlock

United Kingdom

Min Meng

China

Catherine Merrick

United Kingdom

Jane Messina

United Kingdom

Jerrold Meyer

United States

Andrei Daniel Mihalca

Romania

Noboru Minakawa

Japan

Tiago Mineo

Brazil

Paul Mireji

Kenya

Ladslaus Mnyone

Tanzania

Norhayati Moktar

Malaysia

Ricardo Molina

Spain

David Molyneux

United Kingdom

Nadia Monesi

Brazil

Sarah Moore

United Kingdom

Osama Mostafa

Saudi Arabia

Karine Mouline

France

Kate Mounsey

Australia

Catherine Moyes

United Kingdom

Shandala Msangi

Tanzania

Grace Mulcahy

Ireland

Stephen Munga

Kenya

Maria Anice Mureb Sallum

Brazil

Ephantus Muturi

United States

Joseph Mwangangi

Kenya

Georgina Mwansat

Nigeria

Pauline Mwinzi

Kenya

Soraya Naem

Iran

Minoru Nakao

Japan

Ryo Nakao

Japan

Boniface Namangala

Zambia

Ralf Nauen

Germany

Wasi Ahmad Nazni

Malaysia

Raphael N'guessan

Benin

Pin Nie

China

Martin Nielsen

United States

Kenneth Nilsson

Sweden

Alasdair Nisbet

United Kingdom

Sammy Njenga

Kenya

Debbie Nolder

United Kingdom

Harry Noyes

United Kingdom

Patricia Nuttall

United Kingdom

Philippe Nwane

Cameroon

Marcos Obara

Brazil

Barry Oconnor

United States

Peter Odermatt

Switzerland

Nick Ogden

Canada

Susumu Ohtsuka

Japan

Oivind Oines

Norway

Kenichi Okamoto

United States

Lucy Okell

United Kingdom

Fredros Okumu

Tanzania

Ana Oleaga

Spain

Gaetano Oliva

Italy

Peter Olson

United States

Luis Miguel Ortega-Mora

Spain

Pedro Ortiz

Peru

Yoshio Osada

Japan

Jose A Oteo

Spain

Domenico Otranto

Italy

Johnson Ouma

Kenya

Faye Ousmane

Senegal

Chiranjib Pal

India

Weiqing Pan

China

Rosario Panadero

Spain

Francisco Panzera

Uruguay

Elias Papadopoulos

Greece

Paul Parham

United Kingdom

Antoine Pariselle

Cape Verde

Yoonseong Park

United States

Jigensh Patel

United States

Doug Paton

United Kingdom

Bruce Patterson

United States

Fabiana Paula

Brazil

Christophe Paupy

Gabon

Herve Pelloux

France

Cedric Pennetier

Benin

Adalberto Pérez De León

United States

Dusan Petric

Serbia

Carina Pinheiro

Brazil

Jairo Pinheiro

Brazil

Joao Pinto

Portugal

Evangelia - Theophano Piperaki

Greece

George Poinar

United States

Anil Poudel

United States

Natacha Protopopoff

Tanzania

Concepcion Puerta

Colombia

Martha Quinones

Colombia

Juan David Ramirez

Colombia

Jose Manuel Ramos

Spain

Reda Ramzy

Egypt

Haseeb Randhawa

New Zealand

Sarah Randolph

United Kingdom

Hilary Ranson

United Kingdom

Vincent Raquin

France

Michel Raymond

France

Andrew Read

United States

Paul Ready

United Kingdom

Sarah Reece

United Kingdom

Bobby Reiner

United States

Paul Reiter

France

Justin Remais

United States

Jose Requena

Spain

Filiberto Reyes-Villanueva

Mexico

Vitor Ribeiro

Brazil

Dania Richter

Germany

Scott Ritchie

Australia

Gilles Riveau

Senegal

Annapaola Rizzoli

Italy

Bruno Roatt

Brazil

Ian Robert

United States

Joao Rodrigues

Portugal

Michael Rogan

United Kingdom

David Rollinson

United Kingdom

Gislaine Roma

Brazil

Gustavo Romero

Brazil

Luca Rossi

Italy

Xavier Roura

Spain

Mark Rowland

United Kingdom

Guita Rubinsky Elefant

Brazil

Douglas Rugg

United States

Marilyn Ruiz

United States

Angel Sainz

Spain

Mo Salman

United States

Oscar Salomon

Argentina

André Santos

Brazil

Eloina Santos

Brazil

Fred Santos

Brazil

Renato Santos

Brazil

Hiroshi Sato

Japan

Yukita Sato

Japan

Hirofumi Sawa

Japan

Manuela Schnyder

Switzerland

Richard Schulz

Canada

Ryan Schwarz

United States

Chris Secombes

United Kingdom

Nagila Secundino

Brazil

Renfu Shao

Australia

Yaghya Sharma

India

Jilong Shen

China

Andrew Shinn

United Kingdom

Varda Shkap

Israel

Cornelia Silaghi

Germany

Alon Silberbush

United States

Maria Helena Silva-Filha

Brazil

Frederic Simard

Burkina Faso

Andrea Simkova

Czech Republic

Gustave Simo

Cameroon

Paul Erik Simonsen

Denmark

Steven Singer

United States

Marianne Sinka

United Kingdom

Paiboon Sithithaworn

Thailand

Ole Skovmand

France

Hannah Slater

United Kingdom

Despina Smirlis

Greece

Adrian Smith

United Kingdom

Lucy Smith Paintain

United Kingdom

Rodrigo Soares

Brazil

Ricardo Soares Magalhaes

Australia

Ljiljana Sofronic-Milosavljevic

Serbia

Woon-Mok Sohn

Korea South

Philippe Solano

Burkina Faso

Miroslava Soldánová

Czech Republic

Pradya Somboon

Thailand

Ketty Soteriadou

Greece

Manuel Soto

Spain

Susana Sousa

Portugal

Marta Spakulova

Slovakia

Olivier Andre Ettore Sparagano

United Kingdom

Robert Spear

United States

Benjamin Speich

Switzerland

Eva Spitalska

Slovakia

David Spratt

Australia

Hein Sprong

Netherlands

Simona Stager

Canada

Claire Standley

United States

Dorothee Stanneck

Germany

Sasa Stefanic

Switzerland

Peter Steinmann

Switzerland

Dietmar Steverding

United Kingdom

Roger Stich

United States

Russell Stothard

United Kingdom

Michael Strand

United States

Ellen Stromdahl

United States

Snorre Stuen

Norway

Hiromu Sugiyama

Japan

Michael Sukhdeo

United States

Toshihiko Sunahara

Japan

Xun Suo

China

Sinnathamby Surendran

Sri Lanka

Bernd Sures

Germany

Johari Surin

Malaysia

Ben Sutherland

Canada

Milena Svobodová

Czech Republic

Arno Swart

Netherlands

Din Syafruddin

Indonesia

Yasuhiro Takashima

Japan

Kevin Shyong-Wei Tan

Singapore

Sun Tee Tay

Malaysia

Mark Taylor

United Kingdom

Marta Teixeira

Brazil

John Terblanche

South Africa

Urusa Thaenkham

Thailand

Saravanan Thangamani

United States

Van Thi Phan

Viet Nam

R C Andrew Thompson

Australia

Ellen Tijsse-Klasen

Netherlands

Rina Tilak

India

Rafael Toledo

Spain

Steve Torr

United Kingdom

Baldwyn Torto

Kenya

Pablo Tortosa

Reunion

Harold Townson

United Kingdom

Sylvain G Traoré

Cote D'ivoire

Yara Maria Traub-Cseko

Brazil

Donato Traversa

Italy

Omar Triana

Colombia

Takashi Tsunoda

Japan

Michael Turell

United States

Kevin Tyler

United Kingdom

Tatiana Ueno

Brazil

Samuel Ugbomoiko

Nigeria

Igor Uspensky

Israel

Juerg Utzinger

Switzerland

Anayansi Valderrama

Panama

Glyn Vale

Zimbabwe

Jesus Valenzuela

United States

**Gediminas** Valkiūnas

Lithuania

E.Tellervo Valtonen

Finland

Wim Van Bortel

Sweden

Henk van den Berg

Netherlands

Philippe van den Steen

Belgium

Gert Van der Auwera

Belgium

Tullio Vanzetti

Switzerland

Paulo Eduardo Velho

Brazil

V. Venkatesalu

India

Eva Veronesi

United Kingdom

Dario Vezzani

Argentina

Petr Volf

Czech Republic

Georg von Samson-Himmelstjerna

Germany

Jan Votypka

Czech Republic

Indra Vythilingam

Malaysia

David M. Wagner

United States

Jitra Waikagul

Thailand

Anthony Walker

United Kingdom

David Walker

United States

Martin Walker

United Kingdom

Patrick Walker

United Kingdom

Richard Wall

United Kingdom

Joao Luiz Wanderley

Brazil

Chung-Hsiung Wang

Taiwan

Jianyang Wang

United States

Jun-Yun Wang

China

Samuel Wanji

Cameroon

Nicola Wardrop

United Kingdom

Bonnie Webster

United Kingdom

Susan Welburn

United Kingdom

Christopher Whipps

United States

Michael White

United Kingdom

Stephen Wikel

United States

Craig Wilding

United Kingdom

Richard Wilkerson

United States

David Williams

Australia

Anthony Wilson

United Kingdom

James Wilson

United Kingdom

R Alan Wilson

United Kingdom

Margaret Wirth

United States

Charles Wondji

United Kingdom

Jacklyn Wong

United States

Bo Wu

United States

Lihua Xiao

United States

Rajpal Singh Yadav

Switzerland

Laith Yakob

Australia

Javed Yakoob

Pakistan

Chung-Da Yang

Taiwan

Yu Yang

Australia

Zhaoqing Yang

China

Peiling Yap

Switzerland

Tim Yoshino

United States

Simon Young

United Kingdom

Xinbing Yu

China

Mostafa Zamanian

United States

Noraida Zerpa

Venezuela

Bin Zheng

China

Daibin Zhong

United States

Yi-Biao Zhou

China

Xiaohua Zhu

United States

Xing-Quan Zhu

China

Feng-Cai Zou

China

